# Cancer immunogenomic approach to neoantigen discovery in a checkpoint blockade responsive murine model of oral cavity squamous cell carcinoma

**DOI:** 10.18632/oncotarget.23751

**Published:** 2017-12-28

**Authors:** Paul Zolkind, Dariusz Przybylski, Nemanja Marjanovic, Lan Nguyen, Tianxiang Lin, Tanner Johanns, Anton Alexandrov, Liye Zhou, Clint T. Allen, Alexander P. Miceli, Robert D. Schreiber, Maxim Artyomov, Gavin P. Dunn, Ravindra Uppaluri

**Affiliations:** ^1^ Department of Otolaryngology, Washington University School of Medicine, St. Louis, MO, USA; ^2^ Broad Institute of MIT and Harvard, Cambridge, MA, USA; ^3^ Division of Oncology, Department of Medicine, Washington University School of Medicine, St. Louis, MO, USA; ^4^ Center for Human Immunology and Immunotherapy Programs, Washington University School of Medicine, St. Louis, MO, USA; ^5^ Department of Pathology and Immunology, Washington University School of Medicine, St. Louis, MO, USA; ^6^ Dana-Farber Cancer Institute, Boston, MA, USA; ^7^ Head and Neck Surgery Branch, National Institute on Deafness and Other Communication Disorders, Bethesda, MD, USA; ^8^ Department of Otolaryngology-Head and Neck Surgery, Johns Hopkins School of Medicine, Baltimore, MD, USA; ^9^ Department of Neurological Surgery, Washington University School of Medicine, St. Louis, MO, USA; ^10^ Brigham and Women’s Hospital, Boston, MA, USA

**Keywords:** neoantigen, immunogenomics, head and neck cancer

## Abstract

Head and neck squamous cell carcinomas (HNSCC) are an ideal immunotherapy target due to their high mutation burden and frequent infiltration with lymphocytes. Preclinical models to investigate targeted and combination therapies as well as defining biomarkers to guide treatment represent an important need in the field. Immunogenomics approaches have illuminated the role of mutation-derived tumor neoantigens as potential biomarkers of response to checkpoint blockade as well as representing therapeutic vaccines. Here, we aimed to define a platform for checkpoint and other immunotherapy studies using syngeneic HNSCC cell line models (MOC2 and MOC22), and evaluated the association between mutation burden, predicted neoantigen landscape, infiltrating T cell populations and responsiveness of tumors to anti-PD1 therapy. We defined dramatic hematopoietic cell transcriptomic alterations in the MOC22 anti-PD1 responsive model in both tumor and draining lymph nodes. Using a cancer immunogenomics pipeline and validation with ELISPOT and tetramer analysis, we identified the H-2Kb-restricted ICAM1_P315L_ (mICAM1) as a neoantigen in MOC22. Finally, we demonstrated that mICAM1 vaccination was able to protect against MOC22 tumor development defining mICAM1 as a bona fide neoantigen. Together these data define a pre-clinical HNSCC model system that provides a foundation for future investigations into combination and novel therapeutics.

## INTRODUCTION

Despite advances in surgical techniques, chemotherapeutics and targeted radiation therapy, oral cavity squamous cell carcinoma (OSCC) remains a treatment challenge and still carries a 60% 5-year survival rate [1]. Poor outcomes are due, at least in part, to the lack of robust sensitivity of OSCC to adjuvant therapies [2, 3]. To address this need for novel therapeutic modalities, significant efforts have been directed at better understanding the biology of these tumors to identify targeted treatment approaches. However, recent work to characterize the genomic landscape of head and neck squamous cell carcinomas (HNSCC) by the TCGA and others demonstrated that the majority of these tumors do not possess actionable mutations for targeted therapy [4]. In contrast to small molecule approaches, there has been significant interest in the role of immunotherapies in the treatment of HNSCC based largely on the success of these approaches in other carcinogen-induced cancer types [5, 6]. Indeed, two published clinical trials, Keynote-012 and Checkmate-141, demonstrated clinical benefit with anti-PD1 blocking monoclonal antibody (mAb) checkpoint inhibitors in patients with recurrent or metastatic HNSCC [7, 8]. The response rates to anti-PD1 mAbs in these trials were 13.3% [8] and 20% [7], and the failure to meet endpoints in a pembrolizumab Phase III clinical trial [9] together indicate the need for improved biomarkers to predict and monitor responsiveness to checkpoint inhibition.

Similar to approaches using targeted therapies, it is important to identify biomarkers of responses to checkpoint blockade therapy to determine whether the treatement is “on target”. However, detection of an activated, tumor-specific immune response has been limited by our ability to identify and characterize the anti-tumor targets of the immune system. Recent work applying next generation sequencing to T cell antigen identification, termed “cancer immunogenomics”, has greatly facilitated the search for T cell specific targets recognized by the immune system [10–16]. In particular, the identification of “neoantigens” derived from tumor expressed mutations illustrates a functional genomics approach applied to anti-tumor immune recognition. Because the goal of this approach is to identify translated mutations predicted to bind with high affinity to MHC molecules, it emphasizes the antigenic potential of identified missense alterations instead of the classical “driver versus passenger” hierarchy of inferred biological function. The immunogenomics approach was first applied successfully in preclinical settings [14, 15] and has since been employed in expanded basic and translational contexts [17–19]. Importantly, neoantigen discovery may be particularly relevant in the identification of biomarkers of immunotherapy response and also as individual targets in personalized immune-based treatments [20–22].

In HNSCC, there are several potential types of tumor antigens beyond mutation-derived neoantigens, including tumor-associated antigens, cancer-testis antigens, and viral antigens [23]. However, no studies to date have characterized the neoantigen burden of OSCC tumors or identified how anti-PD1 therapy influences the tumor infiltrating lymphocyte (TIL) population. To this end, we sought to identify and characterize tumor antigens in a preclinical carcinogen induced model of OSCC and investigate the association between infiltrating lymphocytes and checkpoint responsiveness. We previously described a syngeneic murine oral cavity squamous cell carcinoma (MOC) cell line model, including the indolent MOC22 and the aggressive MOC2 [24], which displays high fidelity to human OSCC in biologic behavior and genomic landscape [25]. We first evaluated susceptibility of these models to treatment with anti-PD1 checkpoint blocking immunotherapy. To identify mutation-derived neoantigens in these tumors, we analyzed whole exome sequence data [25] of each tumor and applied multiple *in silico* prediction algorithms to identify potential H-2K^b^ and H-2D^b^ restricted neoepitopes. Next, we confirmed the presence of antigen-specific T cells using IFNγ ELISPOT and dual-color tetramer analysis. Finally, we confirmed the therapeutic efficacy of neoantigen immunization in a prophylactic vaccine model. Thus, our proof-of-concept approach of neoantigen discovery and validation in preclinical OSCC contexts demonstrates the potential utility of this paradigm in human HNSCC.

## RESULTS

### Anti PD-1 checkpoint blockade responses

**Figure 1:**
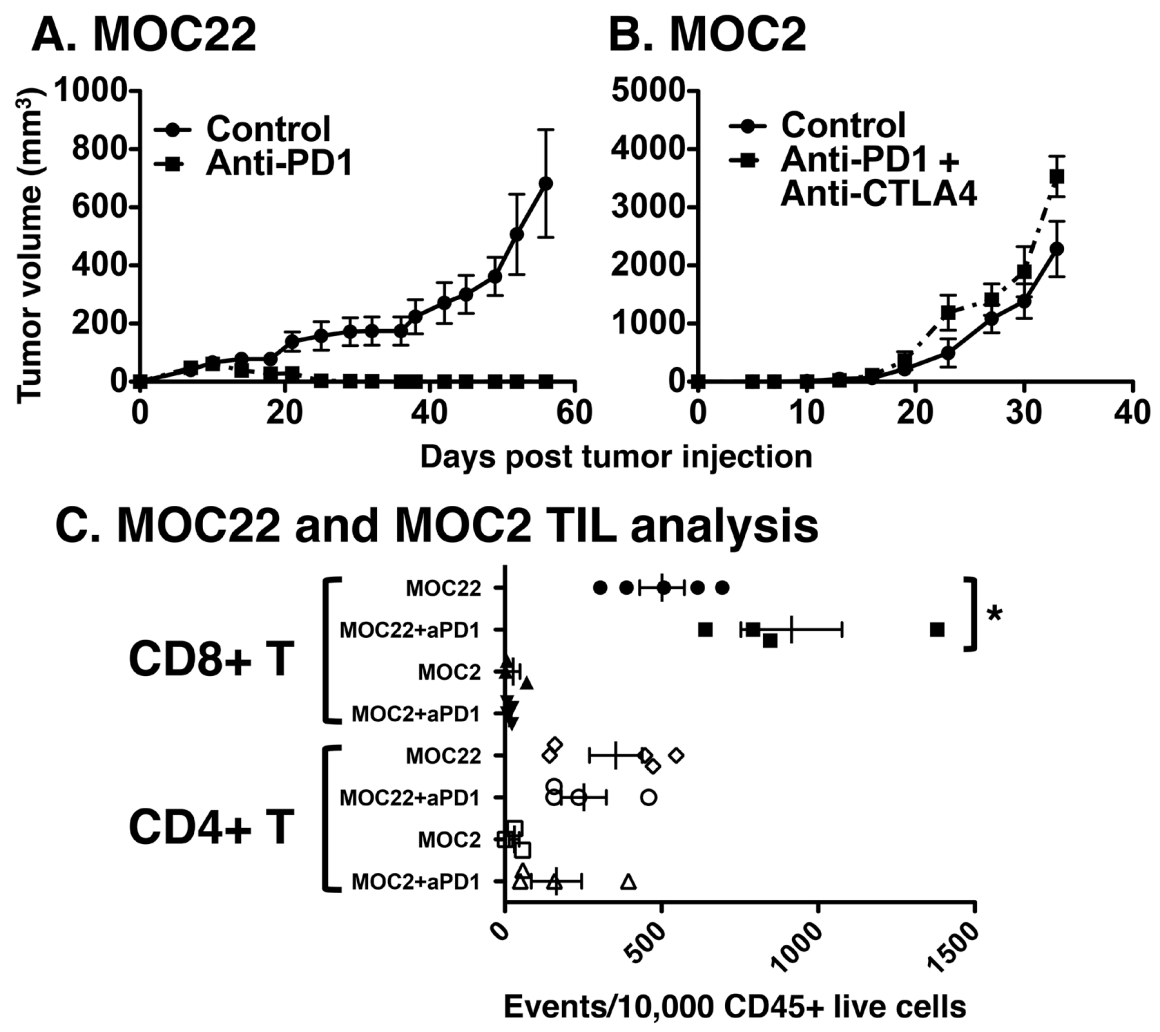
Responsiveness of **(A)** MOC22 to anti-PD1 and **(B)** MOC2 to combination anti-PD1/anti-CTLA4 therapy. Indicated tumor lines were injected at day 0, control or checkpoint targeting antibody therapy was administered on days 3, 6, and 9 and mice were monitored for tumor growth twice weekly. Filled circles are control treated and filled squares represent depleting antibody treated tumors. **(C)** Tumor infiltrating lymphocyte analysis of MOC2 and MOC22 treated with control or anti-PD1 blocking monoclonal antibodies. Tumors were harvested on day 12 post transplant from the indicated tumor bearing mice treated as in (A) and single cell suspensions were analyzed for CD8^+^ or CD4^+^ T cell infiltration normalized to CD45^+^ events (per 10,000 collected, ^*^p<0.05).

To identify an immunogenic preclinical model of OSCC, we investigated the response to checkpoint blockade of two syngeneic tumor cell lines — MOC2 and MOC22 — which grow progressively in wild-type C57BL/6 mice. Anti-PD1 treatment of MOC22 tumors resulted in consistent rejection typically complete by day 35 (Figure 1A). However, MOC2 tumors did not respond to anti-PD1 monotherapy (not shown) or to combination anti-PD1/anti-CTLA4 therapy and grew progressively with rapid growth kinetics (Figure 1B).

To determine whether the distinct response phenotypes of MOC22 and MOC2 correlated with differences in the immune microenvironment, we characterized the tumor immune infiltrate in the absence and presence of anti-PD1 treatment. Relative frequencies of intratumoral CD4^+^ and CD8^+^ T cells were analyzed with and without anti-PD1 treatment at 12 days post-transplant when tumors were at their largest size in MOC22 before the onset of rejection. With control antibody treatment, MOC22 tumors demonstrated significantly higher infiltrating CD8^+^ and CD4^+^ T cells compared to MOC2 tumors (p= 0.0013, p=0.0143, respectively). Whereas MOC22 harbored a significant increase in CD8^+^ T cell infiltration with anti-PD1 treatment (p=0.0195), no difference in the CD8^+^ T cell population was observed in MOC2 following checkpoint blockade treatment (p=0.2924) (Figure 1C). Notably, anti-PD1 treatment did not significantly alter CD4^+^ T cell infiltration in either tumor (MOC22 p= 0.2015, MOC2 p=0.1104).

### Anti-PD1 induced transcriptome alterations in tumor and lymph node immune populations

To characterize transcriptomic alterations in tumor infiltrating hematopoietic cells due to checkpoint blockade by anti-PD1 in MOC22, we used a population RNA-Seq approach to interrogate the transcriptome in mice bearing day 17 tumors. We focused on this timepoint as day 17 typically aligns with the onset of tumor rejection in the anti-PD1 treated cohort. We sorted CD45^+^ hematopoietic cells from non-hematopoietic CD45^-^ cells and performed population RNA-Seq analysis in the CD45^+^ cells in control versus anti-PD1 mice bearing MOC22. Consistent with the FACS data in Figure 1 but distinct from the lymph nodes, CD8a transcripts were significantly increased in the tumor infiltrating immune cells. Other relevant transcripts that were significantly induced in the tumor CD45^+^ cells include Bhlhe40, Eomes S1PR1, TIM3 and CX3CR1.

**Figure 2:**
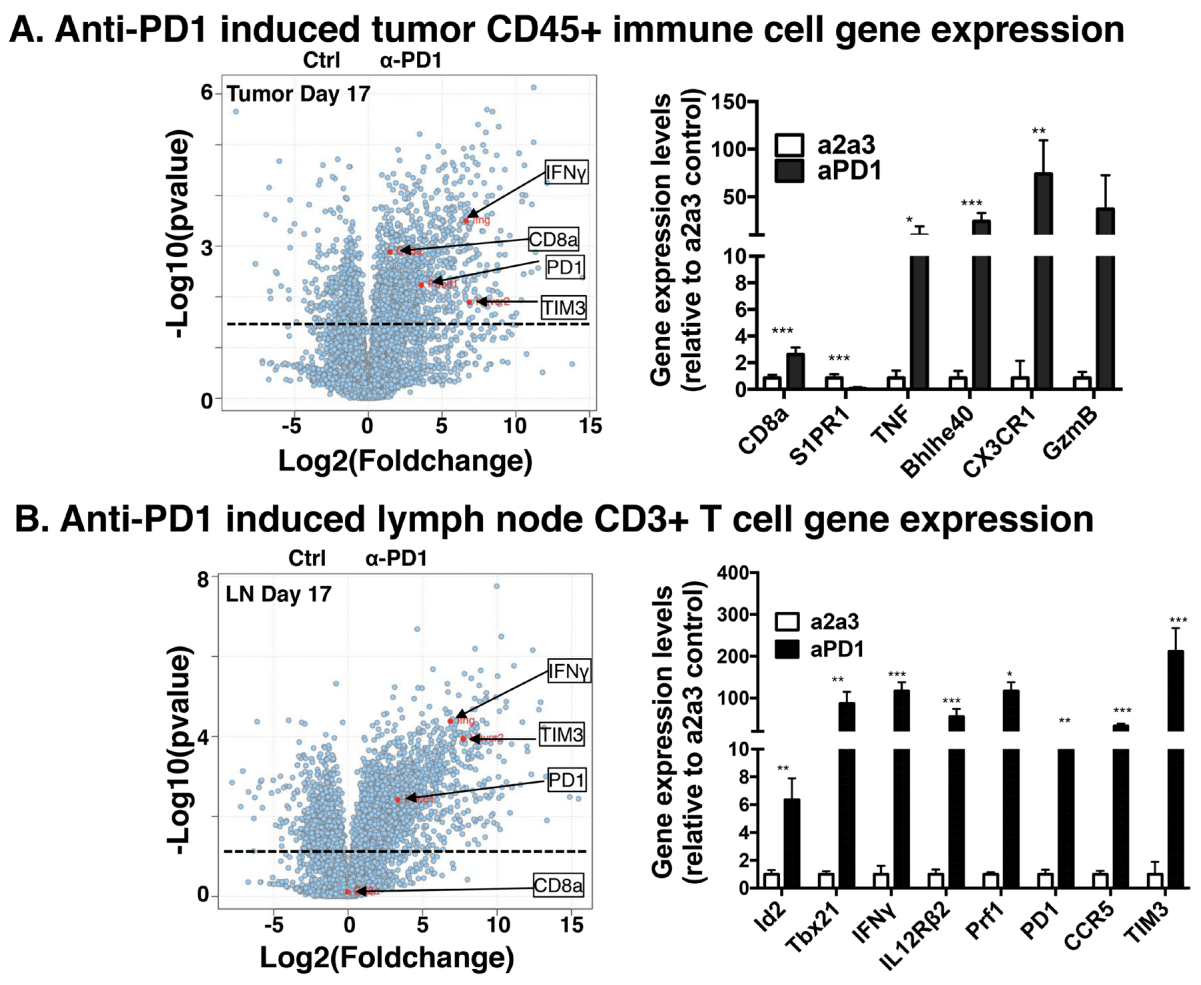
Population RNA-Seq gene expression changes in MOC22 treated with anti-PD1 in tumor microenvironment and draining lymph node MOC22 tumor bearing mice were treated as in 1A and TIL or draining lymph nodes were harvested at day 17, sorted for CD45^+^ immune cells or CD3^+^ T cells, respectively, and subjected to RNA-Seq analysis. **(A)** Intratumoral anti-PD1 induced gene expression changes in D17 MOC22 CD45^+^ hematopoietic populations. Volcano plot on left with specific transcripts highlighted. The bar graph on right shows select relevant transcripts. **(B)** Volcano plot of gene expression changes in draining lymph node CD3^+^ T cells on day 17 post transplant. Highlighted are specific transcripts including IFNγ, TIM3, PD1, and CD8a. The bar graph on right shows select relevant transcripts (^*^=p<0.05, ^**^p<0.01 and ^***^p<0.001).

We next interrogated immune cell populations in draining lymph nodes on days 11, 14 and 17 using a population RNA-Seq approach. These analyses revealed dramatic changes in the draining lymph node CD3^+^ T cell transcriptome with many immunologically relevant targets showing maximal expression at day 17 (Figure 2B). Transcript families that were upregulated with anti-PD1 treatment included transcription factors associated with activated CD4^+^ and CD8^+^ T cell responses (Id2, Id3, Tbx21, Tcf7, Myc, and others), cytokines and receptors involved in T cell proliferation, differentiation and cytolytic activity (IFNγ, TNF, IL7R, IL12Rβ2, Prf1 and GzmB) and surface receptors in lymphocyte migration, adhesion and immune checkpoints (CXCR3, CCR5, CXCR6, Sell, TIM3 and PD1) (Supplementary Figure 1). Interestingly, we did not detect increased CD8 T cell transcripts in the lymph node. These transcriptomic changes were significantly increased in total CD3^+^ T cells in draining lymph nodes on day 17 compared to control antibody treated mice and showed progressive temporal induction/accumulation from day 11 to day 17. Although the overall pattern of increased induction of the distinct families was similar between the tumor milieu and draining lymph nodes, statistical significance was not achieved in several transcripts in the tumor (GzmB, IL7R, TNF, PRF1) and likely reflects the CD3^+^ T cell sort in the lymph node versus the total CD45^+^ hematopoietic sort in the tumor. Together these data show dramatic alterations in immune cell programs induced by anti-PD1 therapy.

### *In silico* neoantigen prediction

**Figure 3:**
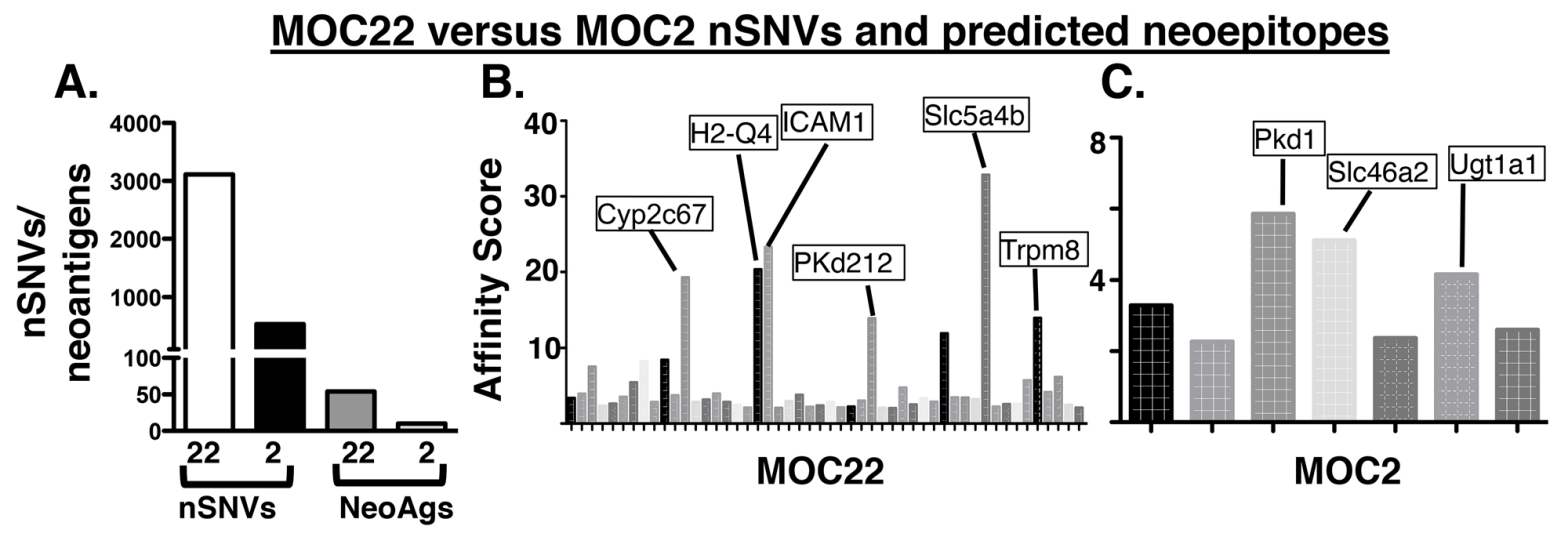
**(A)** Mutation burden of MOC22 and MOC2 and predicted neoantigen burden (IC_50_<50nM) in each tumor. **(B, C)** Manhattan plot of affinity score (1/IC_50_)^*^100 of top candidate neoantigens in MOC2 and MOC22. Labeled are the selected highest predicted binding affinity candidate neoantigens in each tumor cell line.

To determine the potential antigen targets recognized in the checkpoint blockade setting [13], we characterized the mutation burden and subsequent neoantigen landscape of each tumor using an immunogenomics approach. As we have previously described [25], MOC22 has 3,111 nonsynonymous single nucleotide variants (nsSNVs) whereas MOC2 has only 534 (Figure 3A). We then applied an *in silico* bioinformatics pipeline to detect mutation-derived tumor neoantigens in which translated mutant peptides are prioritized based on the predicted affinity to bind to MHC class I in a similar manner as recent studies [13, 16]. Using this approach, we assigned each predicted neoepitope a binding affinity score derived from the average scores of five prediction algorithms. Setting a cutoff of IC_50_ < 500 nM, MOC22 had 762 predicted neoantigens and MOC2 had 109. Applying an even stricter cutoff of 50 nM reduced predicted neoantigens to 50 for MOC22 and 7 for MOC2 (Figure 3B, 3C, Supplementary Tables 1 and 2). To further narrow the list of candidate antigens to only those present in the transcriptome, we then screened a more select list of even higher affinity candidates (IC_50_ <25nM) by targeted PCR and Sanger Sequencing to eliminate non-expressed gene products (data not shown). This approach revealed four high affinity candidates in MOC22 including mutated ICAM-1, H2-Q4, Chst15 and one candidate in MOC2, mutated Pkd1.

### *In vitro* validation

**Figure 4:**
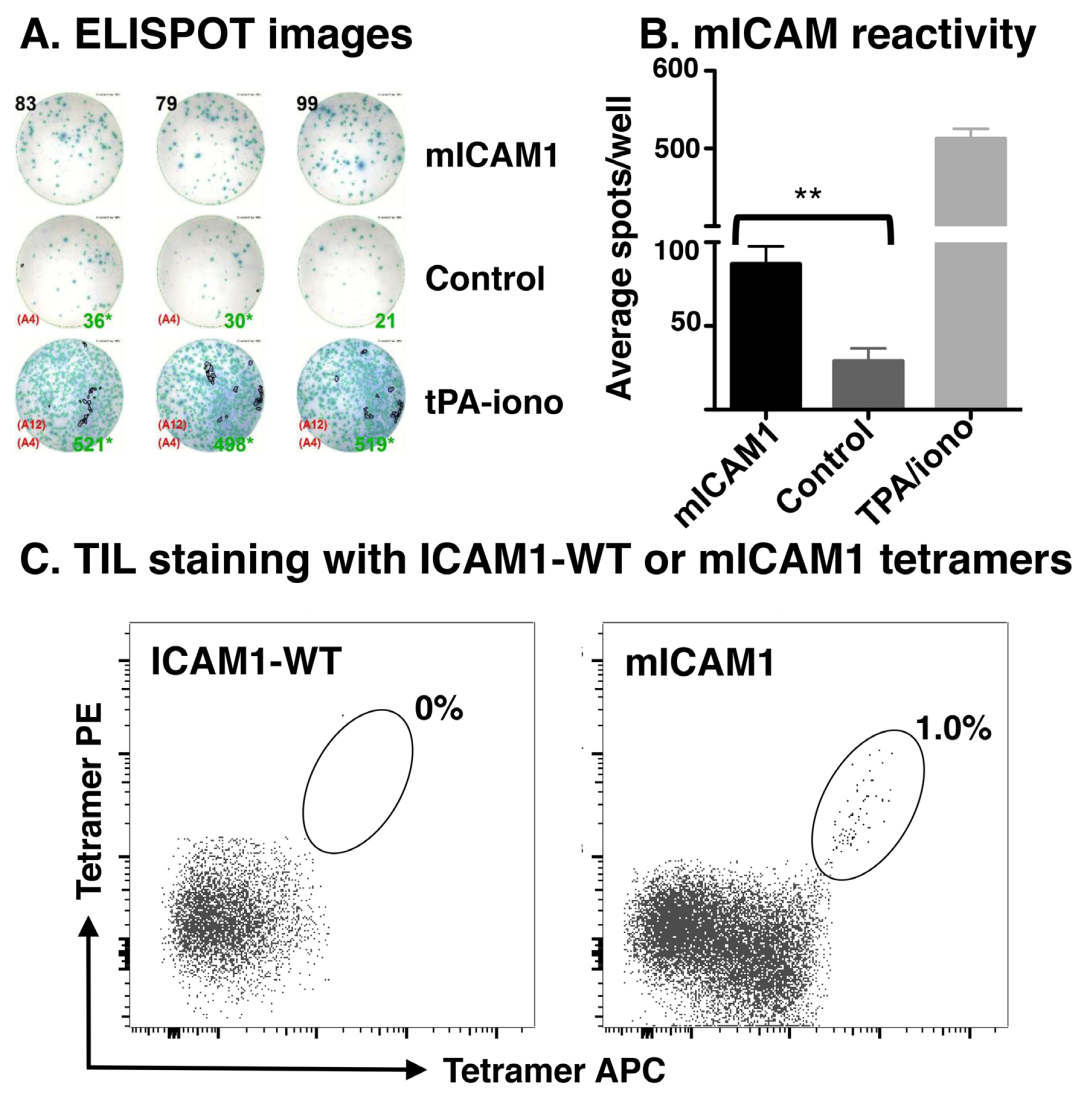
Detection of neoantigen-specific TIL **(A)** Representative images of the IFNγ ELISPOT plate demonstrating TIL reactivity to mICAM1 but not other predicted neoantigens (control including Chst15 and H2-Q4). Tumor infiltrating lymphocytes were isolated from growing MOC22 tumors on day 12, expanded in IL2 media (100U/mL in R10), and plated overnight with 25,000 TIL, 100,000 radiated APCs, and 1μM peptide per well. IFNγ production was assessed the following day by ELISPOT. **(B)** Bar graph quantifying results of IFNγ ELISPOT (^**^p<0.01). **(C)** Detection of mutant ICAM1 neoantigen-specific intratumoral CD8^+^ T cells by dual-labeled tetramer staining. Left panel shows control tetramer with wild type peptide sequence and right panel is with mICAM1 peptide.

Because MOC22 (1) displayed significant CD8^+^ T cell responses, (2) harbored an array of predicted neoantigen targets, and (3) exhibited robust therapeutic responses to anti-PD-1 therapy, we explored whether predicted neoantigen targets were indeed immunogenic. We performed IFNγ ELISPOT with isolated tumor infiltrating lymphocytes (TIL) from established subcutaneously implanted tumors. TIL isolated from MOC22 demonstrated increased activation following stimulation with the H-2K^b^-restricted ICAM1 P315L peptide (referred to as mICAM1; p= 0.0008; Figure 4A, 4B). This approach was attempted with MOC2 as well, but there were insufficient number of CD8^+^ T cells for ELISPOT (data not shown).

Following identification of mICAM1 as a neoantigen candidate for T cells infiltrating MOC22, we next examined whether we could detect antigen-specific T cells ex vivo from the growing tumor using tetramer analysis. TIL isolated from established MOC22 tumors were stained with mICAM1 or control tetramers and analyzed by flow cytometry (Figure 4C). Together, these data demonstrate that MOC22 tumors are infiltrated with T cells that recognize the tumor-derived mICAM1 neoantigen.

### Vaccination

**Figure 5:**
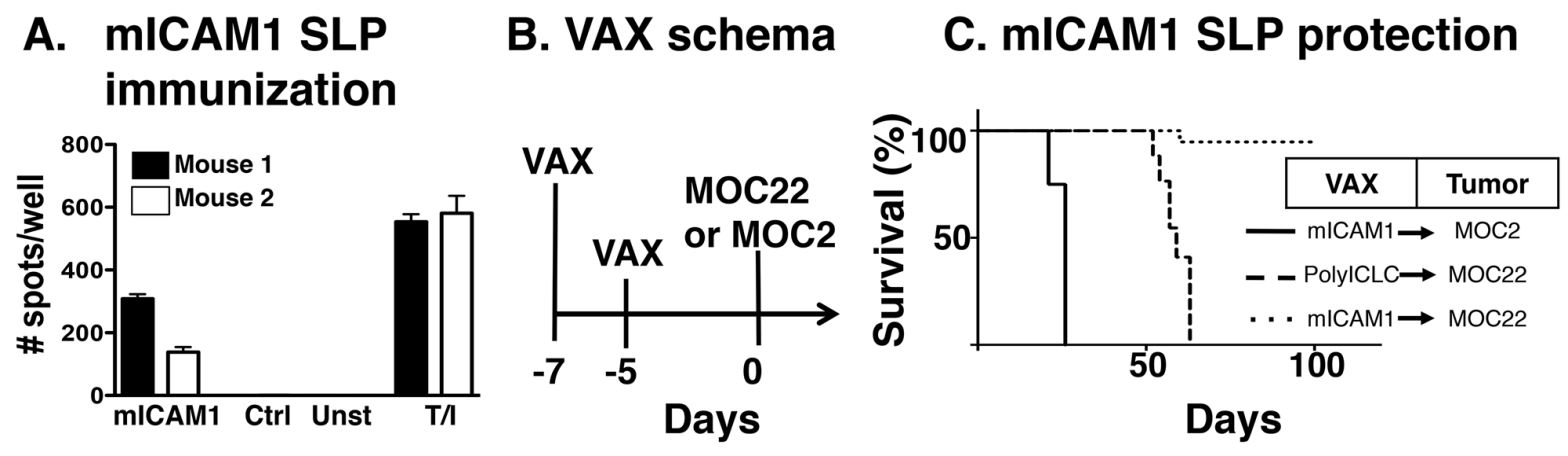
Preventative mICAM1 synthetic long peptide (SLP) vaccination **(A)** Validation of mICAM1 SLP vaccine to generate mICAM1-specific T cell responses. Mice were immunized with mICAM1 SLP or control polyIC:LC alone and spleens were harvested 7 days post-vaccination. Splenocytes (100,000 cells/well) were plated with 1μM short peptide and evaluated for IFNγ production by ELISPOT (T/I is control with TPA and ionomycin stimulation). **(B)** Prophylactic vaccination study design with immunization on days -7 and -5 followed by tumor challenge on day 0. **(C)** Kaplan-Meier survival curve for mICAM1 preventative vaccination. Mice were immunized with PolyIC alone or in combination with mICAM1 SLP and challenged with MOC2 (solid line with mICAM1 vaccine) or MOC22 (dashed line polyIC:LC control or dotted line with mICAM1). This is representative of two independent experiments with the same MOC22 result (n=4 mice for each group).

Finally, to establish whether mICAM1 neoantigen-specific T cells alone can induce tumor rejection of mICAM1-expressing MOC22 cells, we tested the hypothesis that prophylactic vaccination with mICAM1 peptides would protect mice from the growth of MOC22 in the absence of checkpoint blockade immunotherapy (Figure 5A). To establish the efficacy of our vaccination modality to induce mICAM-specific immune responses, mice were immunized mice with two doses of a 28 amino acid synthetic long peptide (SLP) containing the mICAM1 neoantigen together with adjuvant PolyIC:LC. Splenocytes from immunized mice demonstrated significant IFNγ stimulation when cultured with mICAM-1 peptide but not control peptides, demonstrating the immunogenicity of the mICAM-1 SLP. Having established that mICAM-1 SLP plus PolyIC:LC produces antigen specific immunity, we tested the capacity of mICAM-1 vaccination to prevent MOC22 tumor growth by vaccinating mice twice within 1 week prior to tumor implantation (Figure 5B). In mice vaccinated with control poly-ICLC, all MOC22 bearing mice displayed progressively growing tumors. Mutant ICAM1 vaccination did not protect mice from challenge with the unrelated MOC2 tumor cell line. However, MOC22 tumors were rejected in 80% of the mice vaccinated with mICAM1 (Figure 5C). Together, these data show that mICAM1-specific immunity alone can induce the rejection of mICAM-1-expressing MOC22 tumors and validate that mICAM-1 is a bona fide neoantigen capable of tumor protection.

## DISCUSSION

Immunogenomic approaches have dramatically advanced our capacity for neoantigen identification and defined their role as key targets of immunotherapies, as biomarkers of response and, as confirmed in recent studies, their potential as vaccines [26–28]. In addition, several recent studies identified that checkpoint inhibitor-induced tumor rejection is mediated by neoantigen-specific T cells [12, 13]. As carcinogen induced OSCCs have a significant mutation burden, a finding correlated with increased neoantigen loads, this tumor type represents an ideal immunotherapy target. Despite this potential, Phase III data in recurrent, and/or metastatic head and neck cancers shows low response rates with nivolumab and a failure to meet survival endpoints with pembrolizumab. Thus, there is a pressing need to identify OSCC model systems with defined immunobiology in order to better leverage immunotherapeutic modalities in this disease setting. To define a robust pre-clinical system for studying immune based treatments in OSCC, we first delineated checkpoint responses in our syngeneic model system and identified both responsive (MOC22) and resistant (MOC2) models. Transcriptomic approaches provided a window into the temporal and spatial (primary tumor versus lymph node) gene expression changes induced by anti-PD1 blockade in MOC22. We then applied an immunogenomics pipeline and defined the mICAM1 epitope as a neoantigen in MOC22 and demonstrated the potential for personalized antigen specific vaccination in mice bearing this tumor. Thus, these data identify a robust pre-clinical platform with defined responses and biology for further investigating checkpoint and neoantigen specific OSCC studies.

Immunogenomics based approaches were first defined in mouse sarcoma and melanoma models and have since been extended to brain tumors [29]. Broadly speaking, there are manifold applications of neoantigen discovery in cancer—as targets, as biomarkers, and as probes to study the basic immunobiology of T cell responses to endogenous tumor antigens [23]. We anticipate that these approaches will all be highly germane to head and neck cancer. Our work here represents the first study to define both checkpoint and neoantigen specific responses in an OSCC model. Specifically, the immunogenic mICAM neoantigen was detected in both spontaneous immune responses and also was a therapeutically relevant vaccine target in protective settings. Ongoing work in our OSCC models is directed at elucidating the most effective methodologies to vaccinate in therapeutic settings and at investigating combination approaches in the MOC2 tumor that is persistently unresponsive to immunotherapies.

The MOC models were generated in order to establish syngeneic carcinogen induced platforms to investigate host responses to OSCC tumor challenge. Our work highlights key biological observations that need to be clarified at a more granular level. First, our early work established the aggressive biology of the MOC2 tumor and identified it as a T cell “non-inflamed” model [30]. Here, we confirmed these findings and extended data to show that MOC22 contains significant numbers of intratumoral CD8^+^ T cells. Similar to findings in anti-PD1 treated melanoma samples, anti-PD1 treated MOC22 displayed an increase in intratumoral CD8^+^ T cells [31]. There are multiple mechanisms which may contribute to the lack of response to anti-PD1 in MOC2 and these include in part, a lack of high affinity neoantigens and a limited CD8^+^ T cell infiltrate. Ongoing work will be directed at understanding the basis for the poor immunogenicity seen in the MOC2 system.

Second, deep transcriptomic analysis showed significant alterations in global effector/memory, chemotactic and checkpoint programs. Previous analyses of transcriptomic data from antigen specific T cells in a checkpoint responsive murine sarcoma model have defined alterations in metabolic, signaling and T cell effector pathways after treatment with anti-PD1, anti-CTLA-4 or combination therapy [13]. More recently, transcriptomic comparison of CD4^+^ T cells at baseline and antigen specific CD4^+^ T cells after neoantigen vaccination in melanoma patients revealed significant induction of effector and memory T cell gene expression programs including Tbx21 [26]. Herein, we also observed Tbx21 induction in the lymph node CD3^+^ T cell compartment in addition to other effector and memory programs. Importantly, our analysis defines a temporal progression of these changes with maximal induction values for many programs at the day 17 time point within the lymph node. Future work will address the evolution of these changes in gene expression modules both temporally and also spatially with respect to distinct anatomic regions of the tumor and secondary lymph node sites.

The promise of personalized neoantigen vaccine approaches is at an early stage with exciting early trial data in melanoma patients [28, 32]. Our definition of the mutant ICAM1 neoantigen for MOC22 will allow OSCC specific studies. Our current neoantigen biomarker work is aimed at defining mICAM1 specific responses in other immunotherapeutic approaches and in defining optimal therapeutic vaccination schema including rational combination strategies in established tumor settings.

## MATERIALS AND METHODS

### Mice

C57BL/6 mice were purchased from Taconic. All *in vivo* experiments used 8-12 week old female mice housed in a pathogen-free animal facility and all experiments performed were approved by the AAALAC accredited Animal Studies committee of Washington University in St. Louis.

### Tumor transplantation

MOC cell lines were generated, characterized and propagated as previously described [24]. MOC cell lines were cultured in IMDM/Hams-F12 with 5% FCS. For transplantation, cells were washed extensively and resuspended in endotoxin-free PBS (Fisher) and injected subcutaneously into the flanks of mice. Tumor growth was monitored biweekly and tumor volume was calculated based on the formula (L X W^2^)/2.

### Checkpoint blockade experiments

MOC2 and MOC22 cell lines were transplanted into the subcutaneous flank of mice as above. Mice were treated with intraperitoneal injection of anti-PD1 (Clone RMP1-14, Leinco, 250 micrograms) or control antibody (anti-2a3, Leinco, 250 micrograms) on days 3, 6, 9 after transplant. Tumor growth was monitored as above and comparison of the two cohorts was made using two-way ANOVA.

### TIL analysis

MOC22 tumor bearing mice were treated with control or anti-PD1 antibody as above. Day 12 tumors were harvested and processed into single cell suspensions. Cells were stained with Zombie NIR (live/dead cells, BioLegend), CD45 (PerCP-Cy5.5, BioLegend), CD3 (FITC, BioLegend), CD4 (APC, BioLegend), and CD8 (PE, BioLegend) and analyzed on a Fortessa Flow Cytometer. Comparison groups were compared using Student’s t-test.

### Population RNA-Seq analysis

MOC22 tumor bearing mice were treated with control or anti-PD1 antibody as above. Draining lymph nodes (days 11, 14 and 17) or tumor (day 17) were harvested. Single cell suspensions of lymph node cells gated on CD3^+^ or CD3^-^ populations were then FACS sorted, immediately spun down and stored in TCL buffer (Qiagen) snap frozen at -80 C. Tumor cell suspensions were similarly sorted into live CD45^+^ or CD45^-^ cell populations. Samples were processed using SmartSeq 2 protocol (Clontech, Mountain View, CA) with modifications [33]. cDNA generated with Smartseq 2 protocol was converted to Illumina seq libraries using Nextera (Illumina, San Diego, CA). Samples were sequenced on Illumina Nextseq. RNA-Seq reads were aligned to the mouse reference genome with Bowtie 2 [34]. Expression levels were quantified in TPM using RSEM [35]. Differential expression was computed using *t* test after log transformation of expression data. The multiple hypothesis testing correction was computed with Benjamini-Hochberg method [36].

### Neoepitope prediction

Nonsynonymous coding variants for MOC2 and MOC22 [25] were analyzed for potential binding to MHC class I H-2K^b^ or H-2D^b^ molecules using the Stabilized Matrix Method (SSM) algorithm, the Artificial Neural Network (ANN) algorithm, and the NetMHCpan algorithm provided by the Immune Epitope Database and Analysis Resource (http://www.immuneepitope.org) [37–39]. These analyses were used to predict epitope processing and binding affinity and the results are expressed as an affinity value combined with 1/IC_50_ where IC_50_ is the half-maximum inhibitory concentration as has been previously reported [13].

### ELISPOT

Day 12 tumors were resected and cultured in IL-2 (100U/mL) to expand the tumor infiltrating lymphocytes (TIL). After 48 hours, TIL were harvested and selected for live cells (Miltenyi) and incubated at 1.5 x 10^5^ cells with 2.5x10^5^ naïve splenocytes and 1uM peptide (Peptide 2.0) in an ELISPOT plate pre-coated with anti-interferon-γ antibody (Immunospot). ELISPOT was performed per manufacturer’s direction and plates were read in the Immune Monitoring Lab (IML) of the Center for Human Immunology and Immunotherapy Programs (CHIIPS) at Washington University.

### Tetramer staining

Tetramer reagents were produced in the IML of CHIIPS at Washington University. Briefly, recombinant H-2K^b^ and β2-microglobulin were produced in BL21-CodonPlus (DE3)-RIPL *Escherichia coli* (Agilent) and purified from inclusion bodies by size-exclusion FPLC as previously described [40]. UV-mediated exchange of candidate epitopes generated peptide-specific monomers which were multimerized by streptavidin-conjugated PE or APC as previously described [35]. TIL were stained with peptide-MHC I tetramers for 15 minutes at 37°C and then with CD8α-FITC, CD45-PerCP-Cy5.5, and Zombie NIR Fixable Viability Kit and analyzed on a BD Fortessa Flow Cytometer.

### Synthetic long peptide (SLP) vaccination

Immunizations were performed by injecting mICAM1 synthetic long peptide (DQILETQRTLTVYNFSALVLTLSQLEVS, 50 micrograms, Peptide 2.0) with PolyIC:LC (Invivogen, 100 micrograms) or PolyIC:LC alone into the subcutaneous flank on days -7 and -5 and tumor challenge was performed on day 0. Tumor growth was monitored as above.

## SUPPLEMENTARY MATERIALS FIGURE AND TABLES






