# Supplementary material for: Cancer immunogenomic approach to neoantigen discovery in a checkpoint blockade responsive murine model of oral cavity squamous cell carcinoma
Source: Oncotarget. 2017 Dec 28;9(3):4109–19. doi: 10.18632/oncotarget.23751 (PMC5790525; doi:10.18632/oncotarget.23751)
Supplement: Supplementary file 1 [file oncotarget-09-4109-s001.pdf]

Cancer immunogenomic approach to neoantigen discovery in a checkpoint blockade responsive murine model of oral cavity squamous cell carcinoma

SUPPLEMENTARY MATERIALS

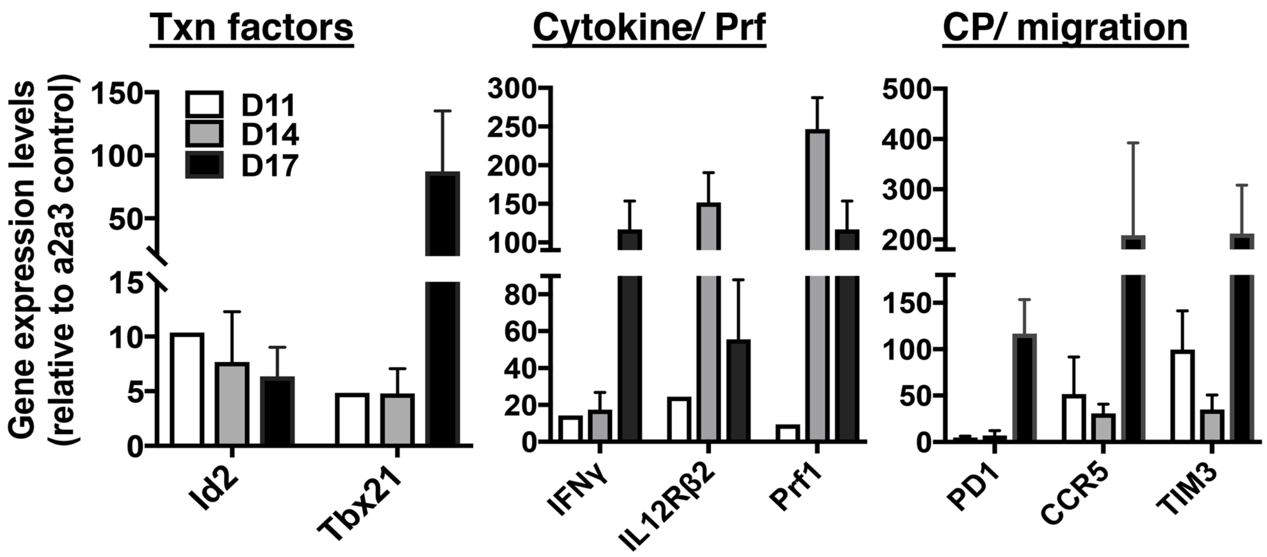

Supplementary Figure 1: Anti-PD1 induced temporal gene expression in LN CD3+ T cells.

Supplementary Table 1: MOC2 Predicted Kb Neoantigens (IC50 <50nM).

See Supplementary File 1

Supplementary Table 2: MOC22 Predicted Kb Neoantigens (IC50 <50nM).

See Supplementary File 2
